# Quantum Transport through a Quantum Dot Coupled to Majorana Nanowire and Two Ferromagnets with Noncollinear Magnetizations

**DOI:** 10.3390/nano14141210

**Published:** 2024-07-16

**Authors:** Yu-Mei Gao, Yao-Hong Shen, Feng Chi, Zi-Chuan Yi, Li-Ming Liu

**Affiliations:** 1 School of Electronic and Information Engineering, UEST of China, Zhongshan Institute, Zhongshan 528400, China; yumeigao@zsc.edu.cn (Y.-M.G.); yizichuan@zsc.edu.cn (Z.-C.Y.); liulmxps@zsc.edu.cn (L.-M.L.); 2 South China Academy of Advanced Optoelectronics, South China Normal University, Guangzhou 510006, China; 2023024194@m.scnu.edu.cn

**Keywords:** quantum transport, tunnel magnetoresistance, quantum dot, ferromagnetic leads, noncollinear magnetizations, Majorana bound states

## Abstract

We study the electron tunneling (ET) and local Andreev reflection (AR) processes in a quantum dot (QD) coupled to the left and right ferromagnetic leads with noncollinear ferromagnetisms. In particular, we consider that the QD is also side-coupled to a nanowire hosting Majorana bound states (MBSs) at its ends. Our results show that when one mode of the MBSs is coupled simultaneously to both spin-up and spin-down electrons on the QD, the height of the central peak is different from that if the MBS is coupled to only one spin component electrons. The ET and AR conductances, which are mediated by the dot–MBS hybridization, strongly depend on the angle between the left and right magnetic moments in the leads. Interaction between the QD and the MBSs will result in sign change of the angle-dependent tunnel magnetoresistance. This is very different from the case when the QD is coupled to regular fermonic mode, and can be used for detecting the existence of MBSs, a current challenge in condensed matter physics under extensive investigations.

## 1. Introduction

With the rapid development of information technology, there is a growing demand for information storage setups possessing faster response speed, lower energy consumption, and higher integration density. It was reported that the proportion of global direct electricity consumption of information and communication technology (ICT) has risen from 3.9% in 2007 to 4.6% in 2012 and to about 10% in 2022 [1,2]. Moreover, the direct energy consumption of ICT sectors is relatively minor compared to that of energy-intensive sectors such as the steel sector [3]. This is then continuously challenging the control methods and hosting materials of electrons’ spin degree of freedom in addition to their charge counterpart. In this context, much recent work has been devoted to the research subject of noncollinear magnetoelectronics [4,5,6], regarding the manipulation of electron transport by the configuration changes of the magnetic moments in ferromagnetic [7,8,9,10,11], antiferromagnetic [12,13], or ferrimagnetic [14,15] multilayers and tunnel junctions. In metallic ferromagnets, the differences between electronic bands and tunneling rates of electrons for majority and minority spins at the Fermi energy induce spin-dependent mobilities, and the directions of magnetic moments in different ferromagnets are either parallel or antiparallel to each other. In the presence of applied electric fields and weak spin-flip scattering processes, a two-channel resistor model can be applied to explain the transport processes and spin-up and spin-down currents flow in parallel. When the magnetic moments of two ferromagnets are in noncollinear configuration (i.e., neither parallel nor antiparallel), the injected spin currents are also noncollinear and may induce spin states in the ferromagnets or the spacer between them pointing in arbitrary directions [4,5,6,10,11], which is tunable by the device materials and noncollinearity angles. Correspondingly, the spin states prepared in the device and the spin currents transport through the system can be efficiently modulated via the relative angles between the magnetic moments; thus, the instrument is evocatively called spin valves.

Noncollinear ferromagnetic materials bring about unique physical phenomena that are absent in collinear ones, such as the interesting magnetoelectric coupling between the noncollinear spin states and spin spiral order, which may lead to the simultaneous breaking of both spatial inversion and time-reversal symmetries [4,5,11,12,14,16,17]. To make devices even more interesting, there has also been much work on combining noncollinear ferromagnetic materials with quantum dots (QDs), in which many parameters, including the dot energy levels, Coulomb interaction, and electrons’ charge or spin degrees of freedom, can be effectively adjusted [7,8,18,19]. It was shown that in a system with a QD sandwiched between the left and right leads with noncollinear magnetizations (FM-QD-FM), the spin valve effect serves as an effective modulation means of the subtle Kondo resonance, resonant tunneling, and Coulomb blockade phenomena, because the spin-polarized current carried by transport processes is made variable by changing both the spin-polarization and magnetizations of the ferromagnets [7,8,19,20,21,22,23]. The spin-polarization and noncollinearity of the spin valve strongly affect the photon-assisted tunneling and thermoelectric processes when the FM-QD-FM is driven by a high-frequency AC voltage bias [24].

In recent investigations, although as two conventionally antagonistic phenomena, the combination of ferromagnetism and superconductivity has attracted much attention since several intriguing novel effects emerge in the superconductor/ferromagnet heterostructures. For example, it is possible to fabricate multistage deposition-lithography Josepheson tunnel switchable nanojunctions employing the influence of the magnetic material over the superconducting wave function [25], which can be also expandable to ferromagnetic biological nanomaterials [26]. Moreover, it was found that a ferromagnetic nanowire can be driven to a nontrivial chiral topological superconducting state when it is deposited on a spin–orbit coupled superconducting substrate, and then a pair of Majorana bound states (MBSs) may be prepared at the ends of the nanowire [27]. The massless and neutral MBSs are of their own antiparticles and topologically protected. These exotic characteristics enable them to be promising in fault-tolerant quantum computation [28,29,30]. Since the MBSs are realizable in superconductor/ferromagnet heterostructures in the absence of magnetic fields generally required in most other schemes [28], many similar platforms were proposed in recent years, such as unconventional superconductor/ferromagnet heterostructures [31], ferromagnetic wires in proximity to conventional superconductors [32], and ferromagnetic chains with magnetic impurities deposited on top of conventional superconductors [33]. Ferromagnetic metallic nanowires sandwiched between two conventional superconductors in proximity to ferromagnetic insulators were also proposed in theory to prepare the MBSs, a platform free from spin–orbit interaction that relies on the interplay of the supercurrents and exchange fields arising from the ferromagnetic insulators [34,35]. Very recently, it was found that topological superconductors with Majorana flat edge mode can be realized when noncollinear magnetic textures are in contact with the s-wave superconductor [36].

**Figure 1:**
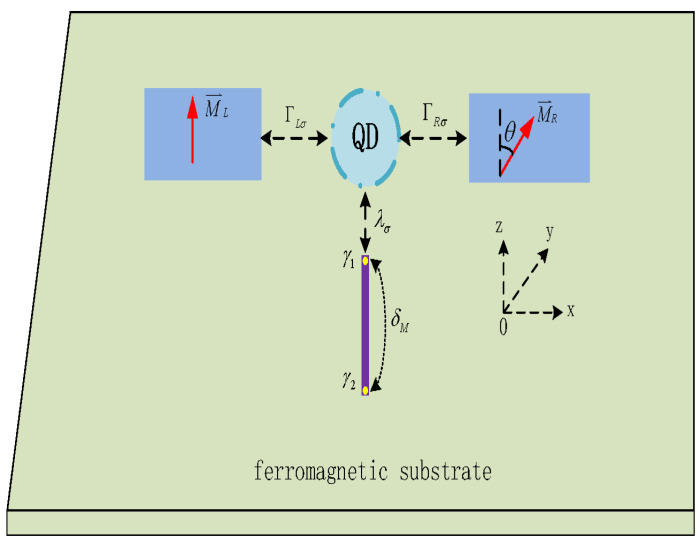
Schematic setup of a quantum dot inserted between two ferromagnetic leads whose magnetic moments form an angle θ. The dot is also side-coupled to a Majorana nanowire (purple band) hosting MBSs (yellow circles), which are denoted individually by $\gamma_{1}$ and $\gamma_{2}$. The MBSs are coupled to the QD with strength of $\lambda_{\sigma }$, and to each other by $\delta_{M}$.

In view of the successful applications of superconductor/ferromagnet heterostructures in preparation and manipulation of MBSs, here, we study quantum transport through a QD simultaneously coupled to a nanowire hosting MBSs and two ferromagnets individually acting as source and drain leads (FM-MQD-FM in Figure 1). One of the motivations of the present paper is to exploit the possibility of detecting the existence of MBSs, an as-yet unsolved research issue [28,37,38,39,40], by means of transport quantities related to the materials’ magnetization, such as the tunnel magnetoresistance (TMR) [20,22,23,41,42,43]. To distinguish the unique properties induced by the presence of MBSs, we compare the results obtained in this FM-MQD-FM with those when the QD is coupled to regular fermonic zero mode, such as another QD, a system denoted by FM-TDQDs-FM. Our numerical results find that the MBSs exert obvious impacts on the linear conductance and TMR from the Andreev reflection (AR) processes, while leaving those from the electron tunneling (ET) processes almost unchanged. The resonant peak values mediated by the MBSs in the presence of ferromagnetic leads are quite different from those when the QD is coupled to normal metallic leads. In particular, both the linear conductance and TMR are sensitive to the relative angles between the leads’ magnetizations. The TMR can even change its sign by the dot–MBS coupling, which is beyond the reach of coupling between QD and regular fermionic mode under the same conditions. This phenomenon may, thus, serve as a detection method for the existence of MBSs.

## 2. Model and Method

The structure studied here is described by the Hamiltonian of $H=H_{QD}+H_{leads}+H_{MNWs}+H_{T}$ [7,8,43,44,45], in which

(1a) $$\begin{array}{ccc} & & H_{QD}=\varepsilon_{d}\sum_{\sigma }d_{\sigma }^{†}d_{\sigma }, \end{array}$$

(1b) $$\begin{array}{ccc} & & H_{leads}=\sum_{\alpha =L,R;k,\sigma }\varepsilon_{\alpha k\sigma }c_{\alpha k\sigma }^{†}c_{\alpha k\sigma }, \end{array}$$

(1c) $$\begin{array}{ccc} & & H_{MNWs}=i\delta_{M}\gamma_{1}\gamma_{2}+\sum_{\sigma }\lambda_{\sigma }\left(d_{\sigma }-d_{\sigma }^{†}\right)\gamma_{1}, \end{array}$$

(1d) $$\begin{array}{ccc} & & H_{T}=\sum_{k,\sigma }\left[V_{kL\sigma }c_{kL\sigma }^{†}d_{\sigma }+V_{kR\sigma }\left(\cos\frac{\theta }{2}c_{kR\sigma }^{†}+\sigma \sin\frac{\theta }{2}c_{kR\bar{\sigma }}^{†}\right)d_{\sigma }+H.c.\right], \end{array}$$

where the creation (annihilation) operator $d_{\sigma }^{†}$$\left(d_{\sigma }\right)$ is for electrons on the QD with spin-independent energy level $\varepsilon_{d}$, which is tunable via gate voltage. The energy of conduction electrons with spin direction σ=↑,↓ and wave vector *k* in lead α is denoted by $\varepsilon_{\alpha k\sigma }=\;\varepsilon_{\alpha k}+\sigma M_{\alpha }$, where $M_{\alpha }$ is the amplitude of the magnetic moment in lead α [7,8]. In the present paper, we assume that the direction of the magnetic moment ${\vec{M}}_{L}$ of the left lead is aligned with the *z* axis, while that of the right lead ${\vec{M}}_{R}$ has a tilted angle θ with respective to ${\vec{M}}_{L}$, forming a noncollinear magnetic configuration [7,8,23,24]. The quantity $\delta_{M}$ in the Hamiltonian $H_{MNWs}$ is the direct hybridization strength between the MBSs with self-conjugate operators $\gamma_{1,2}$, i.e., $\gamma_{i}=\gamma_{i}^{†}\left(i=1,2\right)$, and $\left\{\gamma_{i},\gamma_{j}\right\}=2\delta_{i,j}$ [44,45,46,47,48,49]. Here, we consider that only one mode of the MBSs $\gamma_{1}$ is coupled to the QD with spin-dependent coupling strength $\lambda_{\sigma }$ [45,49,50]. The Hamiltonian $H_{T}$ in Equation (1) describes tunneling between the QD and the leads with amplitude of $V_{k\alpha \sigma }$. To proceed, we make make a unitary transformation to change the Majorana fermion representation to a conventional fermion one [44,46,47,48,49,50], $f=\left(\gamma_{1}+i\gamma_{2}\right)/\sqrt{2}$ and $f^{†}=\left(\gamma_{1}-i\gamma_{2}\right)/\sqrt{2}$. Then, the total Hamiltonian is rewritten in a matrix form to calculate the Green’s functions needed for the electrical current through the QD. On the basis of $\Psi ={\left(d_{↑}^{†},d_{↓},d_{↓}^{†},d_{↑},f^{†},f\right)}^{†}$, the retarded Green’s function is calculated from the Dyson equation method as [44,46,47,48,49,50]

(2) $$\begin{array}{c} G^{r}=g^{r}+g^{r}\Sigma^{r}G^{r}, \end{array}$$

in which $g^{r}$ is the retarded Green’s function of the isolated QD and MBSs and $\Sigma^{r}$ represents the self-energy due to QD–leads and QD–MBS couplings. The diagonal $g^{r}$ is given by

(3) $$\begin{array}{c} g^{r}=\mathrm{diag}{\left(\varepsilon -\varepsilon_{d},\varepsilon +\varepsilon_{d},\varepsilon -\varepsilon_{d},\varepsilon +\varepsilon_{d},\varepsilon +\delta_{M},\varepsilon -\delta_{M}\right)}^{-1}, \end{array}$$

while $\Sigma^{r}$ is

(4) $$\begin{array}{c} \Sigma^{r}=\left[\begin{array}{ccc} \;\;\Sigma_{L}^{r}+\Sigma_{R}^{r} & & \Lambda \\ \;\;\;\;\;\;\Lambda^{T} & & 0 \end{array}\right]. \end{array}$$

The self-energy $\Sigma_{L/R}^{r}$ is contributed from coupling between the QD and the left/right ferromagnetic lead, with [7,23,24,51]

(5) $$\begin{array}{c} \Sigma_{L}^{r}=-\frac{i}{2}\mathrm{diag}\left(\Gamma_{L↑},\Gamma_{L↓},\Gamma_{L↓},\Gamma_{L↑}\right), \end{array}$$

where $\Gamma_{L\sigma }$ is the spin-dependent coupling strength, defined by $\Gamma_{L\sigma }=2\pi \rho_{L\sigma }{\left|V_{kL\sigma }\right|}^{2}$, with $\rho_{L\sigma }$ as the density of states of spin σ band in the left ferromagnetic lead. The matrix elements of $\Sigma_{R}^{r}$ are individually given by [7,24,51] $\Sigma_{R;11}^{r}=\Sigma_{R;44}^{r}=\cos^{2}\frac{\theta }{2}\Gamma_{R↑}+\sin^{2}\frac{\theta }{2}\Gamma_{R↓}$, $\Sigma_{R;22}^{r}=\Sigma_{R;33}^{r}=\cos^{2}\frac{\theta }{2}\Gamma_{R↓}+\sin^{2}\frac{\theta }{2}\Gamma_{R↑}$, $\Sigma_{R;13}^{r}=\Sigma_{R;31}^{r}=\Sigma_{R;24}^{r}=\Sigma_{R;42}^{r}=\frac{\sin\theta }{2}\left(\Gamma_{R↑}-\Gamma_{R↓}\right)$. The coupling strength between the QD and the right lead $\Gamma_{R\sigma }$ is defined similarly to that of $\Gamma_{L\sigma }$, i.e., $\Gamma_{R\sigma }=2\pi \rho_{R\sigma }{\left|V_{kR\sigma }\right|}^{2}$. The self-energy due to the dot–MBS coupling is as follows [46,49,50]:

(6) $$\begin{array}{c} \Lambda =\frac{1}{\sqrt{2}}\left[\begin{array}{ccc} -\lambda_{↑} & & -\lambda_{↑}\\ \;\;\lambda_{↓} & & \lambda_{↓}\\ -\lambda_{↓} & & -\lambda_{↓}\\ \;\;\lambda_{↑} & & \lambda_{↑} \end{array}\right], \end{array}$$

and $\Lambda^{T}$ is the transposed matrix of Λ (here, we assume that $\lambda_{\sigma }$ is real).

To calculate the current flowing through the system, one also needs the lesser Green’s function for the electrons on the QD. We then extract the matrix elements in lines and columns from one to four from the Green’s functions in Equation (2) to form the retarded Green’s function for the QD, i.e., $G^{r}=G^{r}\left(1:4;1:4\right)$. The 4×4 QD lesser Green’s function is then defined as $G^{<}=G^{r}\Sigma^{<}G^{a}$, where $G^{a}={\left(G^{r}\right)}^{†}$ is the advanced Green’s function and $\Sigma^{<}=\Sigma_{L}^{<}+\Sigma_{R}^{<}$ is the lesser self-energy contributed from the two leads. By applying the fluctuation–dissipation theorem, one obtains that $\Sigma_{\alpha }^{<}=F_{\alpha }\left(\Sigma_{\alpha }^{a}-\Sigma_{\alpha }^{r}\right)$ with the advanced self-energy $\Sigma_{\alpha }^{a}={\left(\Sigma_{\alpha }^{r}\right)}^{†}$ and [50,51,52,53,54]

(7) $$\begin{array}{c} F_{\alpha }=\mathrm{diag}\left(f_{\alpha },{\tilde{f}}_{\alpha },f_{\alpha },{\tilde{f}}_{\alpha }\right), \end{array}$$

where $f_{\alpha }=1/\left\{\exp\left[\left(\varepsilon -\mu_{\alpha }\right)/k_{B}T\right]+1\right\}$ and ${\tilde{f}}_{\alpha }=1/\left\{\exp\left[\left(\varepsilon +\mu_{\alpha }\right)/k_{B}T\right]+1\right\}$ are, individually, the electron and hole Fermi distribution functions in lead α. In the above expression, $\mu_{\alpha }$, *T*, and $k_{B}$ denote chemical potential, system temperature, and Boltzmann constant, respectively.

The electronic current $J_{L}$ flowing from the left lead to the QD is obtained in terms of the Green’s functions of $G^{r}$ and $G^{<}$ as [50,51,52,53,54]

(8) $$\begin{array}{c} J_{L}=J_{L↑}+J_{L↓}=\frac{e}{h}\int d\varepsilon {\left[{\left(G\Sigma_{L}\right)}^{<}+H.c.\right]}_{11+33}, \end{array}$$

in which ${\left(G\Sigma_{L}\right)}^{<}=G^{r}\Sigma_{L}^{<}+G^{<}\Sigma_{L}^{a}$, and ${\left[\;\;\right]}_{11+33}={\left[\;\;\right]}_{11}+{\left[\;\;\right]}_{33}$. In the present paper, we consider that an opposite bias *V* is applied between the two leads, $\mu_{L}=-\mu_{R}=eV/2$. In such a case, we have $J_{L}=-J_{R}$, $f_{L}={\tilde{f}}_{R}$, and only ET and local AR processes are allowed to occur [51,53,54,55]. Taking the explicit expressions of the above Green’s functions and self-energies into consideration, the current $J_{L}$ is finally written as

(9) $$\begin{array}{c} J_{L}=\frac{e}{h}\int d\varepsilon \left[T_{ET}\left(\varepsilon \right)+T_{AR}\left(\varepsilon \right)\right]\left(f_{L}-f_{R}\right), \end{array}$$

in which the transmission of ET transport process is [51,53,54]

(10) TET(ε)=(cos2θ2ΓR↑+sin2θ2ΓR↓)(ΓL↑|G11r|2+ΓL↓|G31r|2)+(cos2θ2ΓR↓+sin2θ2ΓR↑)(ΓL↑|G13r|2+ΓL↓|G33r|2)+sinθ(ΓR↑−ΓR↓)Re(ΓL↑G11rG13r*+ΓL↓G33rG31r*),

and

(11) $$\begin{array}{c} T_{AR}\left(\varepsilon \right)=\Gamma_{L↑}(\Gamma_{L↑}|G_{14}^{r}{|}^{2}+\Gamma_{L↓}|G_{34}^{r}{|}^{2})+\Gamma_{L↓}(\Gamma_{L↑}|G_{12}^{r}{|}^{2}+\Gamma_{L↓}|G_{32}^{r}{|}^{2}). \end{array}$$

## 3. Numerical Results

In numerical calculations, the ferromagnetism on the leads is described by the spin-polarization parameter, defined as [7,8,23,24] $P_{\alpha }=\left(\Gamma_{\alpha ↑}-\Gamma_{\alpha ↓}\right)/\left(\Gamma_{\alpha ↑}+\Gamma_{\alpha ↓}\right)$. We then obtain $\Gamma_{\alpha \sigma }=\Gamma_{\alpha }\left(1+\sigma P_{\alpha }\right)$ with $\Gamma_{\alpha }=\left(\Gamma_{\alpha ↑}+\Gamma_{\alpha ↓}\right)$. In the present paper, we consider the case of the two leads made of the same material having the same spin-polarization, i.e., $P_{L}=P_{R}=P$, and set $\Gamma_{L}=\Gamma_{R}=\Gamma \equiv 1$ to be the energy unit. The spin-up and spin-down dot–MBS coupling strengths are also given with the help of a spin-polarization parameter $P_{M}$ as $\lambda_{↑}=\lambda_{0}\left(1-P_{M}\right)$ and $\lambda_{↓}=\lambda_{0}P_{M}$ [45,49]. Moreover, we consider the zero-temperature case, which is favorable for the preparation of MBSs. Therefore, the linear conductances are $G_{ET}=\left(2e^{2}/h\right)\left[T_{ET}\left(\mu \right)\right]$ and $G_{AR}=\left(2e^{2}/h\right)\left[T_{AR}\left(\mu \right)\right]$ [55]. In previous work on a spinless QD coupled to normal metal leads, the conductance peak at zero temperature is suppressed to be half of its quantum value $e^{2}/\left(2h\right)$ by QD–MBS coupling when the QD is on resonance and symmetrically coupled to the leads [44], whereas if the dot is coupled to a regular fermionic zero mode (such as another QD), the conductance is suppressed to be 0 [56,57]. This is strong evidence of the existence of MBSs. One of the main tasks of the present paper is to examine how the conductance is changed when the QD is coupled to ferromagnetic leads, in particular when their magnetic moments are arranged in noncollinear configuration.

**Figure 2:**
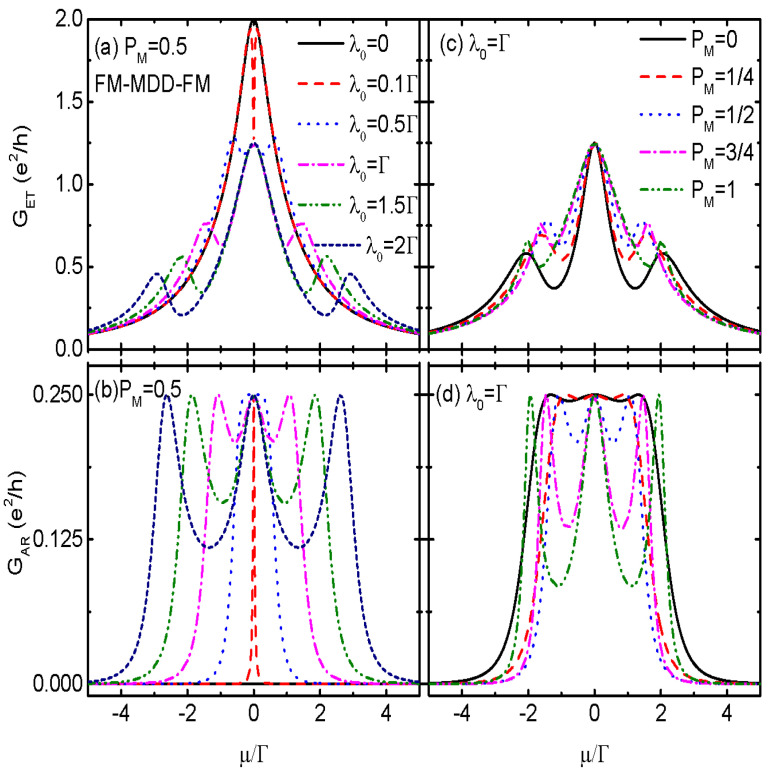
Electron tunneling conductance $G_{ET}$ in (**a**,**c**), Andreev reflection conductance $G_{AR}$ in (**b**,**d**) for the case of FM-MQD-FM as functions of chemical potential μ for different values of dot–MBSs coupling strength $\lambda_{0}$ in (**a**,**b**), and different spin-polarization of the dot–MBSs coupling strength $P_{M}$ in (**c**,**d**). Other parameters in addition to those listed in the figures are spin-polarization of the ferromagnetic leads $P_{L}=P_{R}=P=0.5$, angle between the left and right magnetizations θ=0, MBS–MBS overlap amplitude $\delta_{M}=0$, and dot level $\varepsilon_{d}=0$.

Figure 2 shows $G_{ET}$ and $G_{AR}$ varying with respective to the chemical potential μ under the conditions of θ=0, P=0.5, and $\varepsilon_{d}=0$. The spin-polarization of dot–MBS coupling is fixed to be $P_{M}=0.5$ which means that spin-up and spin-down electrons are coupled to the MBS with equal strength, $\lambda_{↑}=\lambda_{↓}=\lambda_{0}/2$. When the QD is decoupled from the MBSs ($\lambda_{0}=0$), the system becomes FM-QD-FM and the ET conductance is characterized by a single-peak configuration. The central peak value of $G_{ET}\left(\mu =0\right)$ reaches its quantum value $2\;e^{2}/h$, as indicated by the black solid line in Figure 2a. Now the AR process is absent and $G_{AR}=0$, which is shown by the black solid line in Figure 2b [44,55]. Turing on the QD–MBS coupling, $\lambda_{0}\neq 0$, and the system becomes FM-MQD-FM, the conductance peak of $G_{ET}$ in Figure 2a is split and lowered to a fixed value of $1.25\;e^{2}/h$. With increasing $\lambda_{0}$, the peak height of $G_{ET}\left(\mu =0\right)$ remains unchanged, with the two satellite peaks located individually at $\varepsilon_{d}=\pm 2\sqrt{\lambda_{↑}^{2}+\lambda_{↓}^{2}}$. In the presence of dot–MBS coupling, AR process occurs and $G_{AR}$ develops three peaks with equal height of $0.25\;e^{2}/h$. Moreover, the peaks’ positions of $G_{AR}$ and $G_{ET}$ are the same. The height of the central peak in total conductance $G\left(\mu =0\right)=G_{ET}\left(\mu =0\right)+G_{AR}\left(\mu =0\right)=1.5\;e^{2}/h$. Since now electrons of the two spin directions couple to the MBS with equal strength $\lambda_{↑}=\lambda_{↓}$, we have $G_{↑}\left(\mu =0\right)=G_{↓}\left(\mu_{d}=0\right)=0.75\;e^{2}/h$ [45], which is quite different from the case when the MBS is coupled to spinless QD in which $G\left(\mu =0\right)=e^{2}/\left(2h\right)$ [44,55].

In fact, under this simple case, the analytical expression of the spin-dependent total conductance can be obtained as follows:

(12) $$\begin{array}{c} G_{\sigma }=\frac{e^{2}}{h}\Gamma_{\sigma }\frac{\Gamma_{\sigma }\Gamma_{\bar{\sigma }}\lambda_{\bar{\sigma }}^{2}+\left[{\left(\mu -\varepsilon_{d}\right)}^{2}+\Gamma_{\bar{\sigma }}^{2}\right]\lambda_{\sigma }^{2}/2}{\left[{\left(\mu^{-}\varepsilon_{d}\right)}^{2}+\Gamma_{\sigma }^{2}\right]\Gamma_{\bar{\sigma }}\lambda_{\bar{\sigma }}^{2}+\left[{\left(\mu -\varepsilon_{d}\right)}^{2}\right)+\Gamma_{\bar{\sigma }}^{2}]\Gamma_{\sigma }\lambda_{\sigma }^{2}}, \end{array}$$

in which $\Gamma_{\sigma }=\left(\Gamma_{L\sigma }+\Gamma_{R\sigma }\right)/2$. The results presented in Figure 2 are inconsistentwith the analytical expression of $G_{\sigma }$. Note that a similar result was also found in the system of the QD coupled to nonmagnetic leads and Majorana nanowire [45]. In Figure 2c,d we present $G_{ET}$ and $G_{AR}$ as functions of the chemical potential for fixed $\lambda_{0}$ and different values of spin-polarization $P_{M}$. Both of the two conductances remain as triple-peak configurations, and the the peaks’ values at μ=0 are also fixed regardless of the variation of $P_{M}$. As a result of this, the central peak value of the total conductance *G* remains $1.5\;e^{2}/h$ despite the value of coupling strengths between the dot and the two spin component electrons. Under the conditions of strong asymmetric coupling with $P_{M}=0$ and $P_{M}=1$, the values of $\left(\lambda_{↑},\lambda_{↓}\right)$ are individually $\left(\lambda_{0},0\right)$ and $\left(0,\lambda_{0}\right)$, and the MBS couples only to one spin level of the dot. Assuming that spin-σ electrons on the QD are coupled to the MBS, one finds the spin-dependent total conductance $G_{\sigma }\left(\mu =0\right)=e^{2}/\left(2h\right)$ which is independent of the dot level and dot–lead coupling strength, and $G_{\bar{\sigma }}\left(\mu =0\right)=e^{2}\Gamma_{\bar{\sigma }}^{2}/\left[h\left(\varepsilon_{d}^{2}+\Gamma_{\bar{\sigma }}^{2}\right)\right]$, being inconsistentwith the result in FM-QD-FM [8,24].

**Figure 3:**
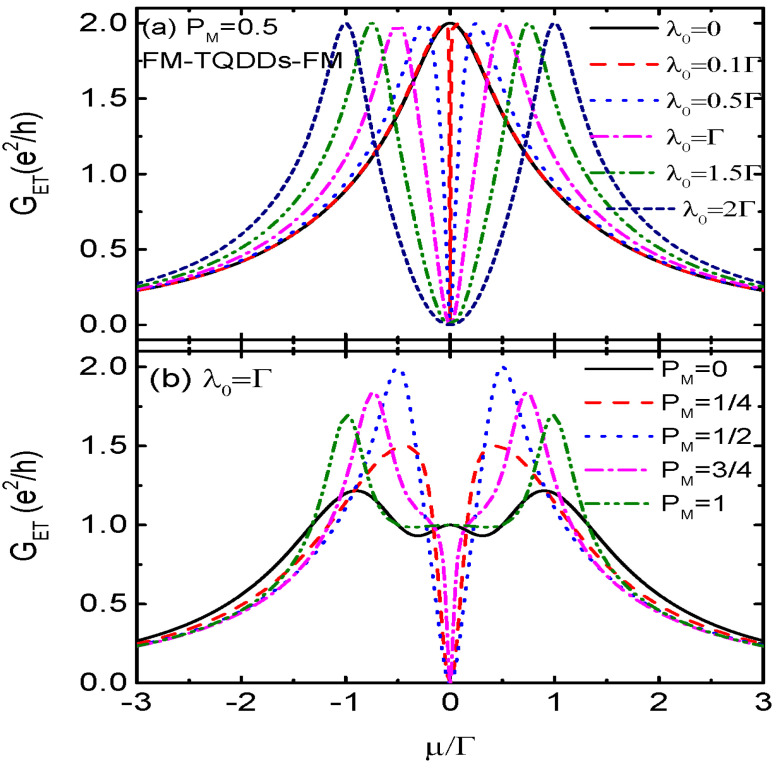
Electron tunneling conductance $G_{ET}$ for the case of FM-TDQDs-FM as a function of chemical potential μ for different values of coupling strength between the dots $\lambda_{0}$ in (**a**), and different spin-polarization of dot–dot coupling strength $P_{M}$ in (**b**). Other parameters are the same as in Figure 2. Note that when the central QD is coupled to another QD behaving as a regular fermion, the AR conductance $G_{AR}\equiv 0$.

To recognize the exotic characteristics in the transport properties brought about by the MBSs, we present in Figure 3 the conductance when the QD is coupled to another dot serving as a regular fermionic zero mode, and forms a T-shaped double QDs (TDQDs) configuration [56,57]. We denote the creation (annihilation) operator, energy level, and coupling strength between the two dots by the same symbols as those of the MBSs for the sake of consistency. The Hamiltonian of the TDQDs is written as follows:

(13) $$\begin{array}{c} H_{TDQDs}=\varepsilon_{d}\sum_{\sigma }d_{\sigma }^{†}d_{\sigma }+\delta_{M}\gamma_{1}^{†}\gamma_{1}+\sum_{\sigma }\lambda_{\sigma }\left(\gamma_{1}^{†}d_{\sigma }+d_{\sigma }^{†}\gamma_{1}\right), \end{array}$$

and the spin-dependent conductance is obtained as

(14) $$\begin{array}{c} G_{\sigma }=\frac{2e^{2}}{h}\frac{\Gamma_{\sigma }^{2}{\left(\mu -\delta_{M}\right)}^{2}}{{\left[\left(\mu -\varepsilon_{d}\right)\left(\mu -\delta_{M}\right)-\lambda_{\sigma }^{2}\right]}^{2}+\Gamma_{\sigma }^{2}{\left(\mu -\delta_{M}\right)}^{2}}, \end{array}$$

which indicates that $G_{\sigma }\left(\mu =0\right)=2\;e^{2}/h$ in the case of $\lambda_{0}=\delta_{M}=0$, as is shown by the black solid line in Figure 3a. When the central QD is coupled to another dot ($\lambda_{0}\neq 0$), the single peak in $G_{\sigma }$ is split into two, positioned at $\mu =\left(\varepsilon_{d}+\delta_{M}\right)/2\pm \sqrt{{\left(\varepsilon_{d}-\delta_{M}\right)}^{2}/4+\lambda_{\sigma }^{2}}$, and the dip at μ=0 is suppressed to be zero. With increasing $\lambda_{0}$, the heights of the two peaks in $G_{\sigma }$ remain unchanged and are shifted toward negative and positive energy regimes, respectively. Note that in these TDQDs, $G_{AR}\equiv 0$ from the AR process is forbidden. These results are totally different from those of FM-MQD-FM presented in Figure 2a [44,55].

**Figure 4:**
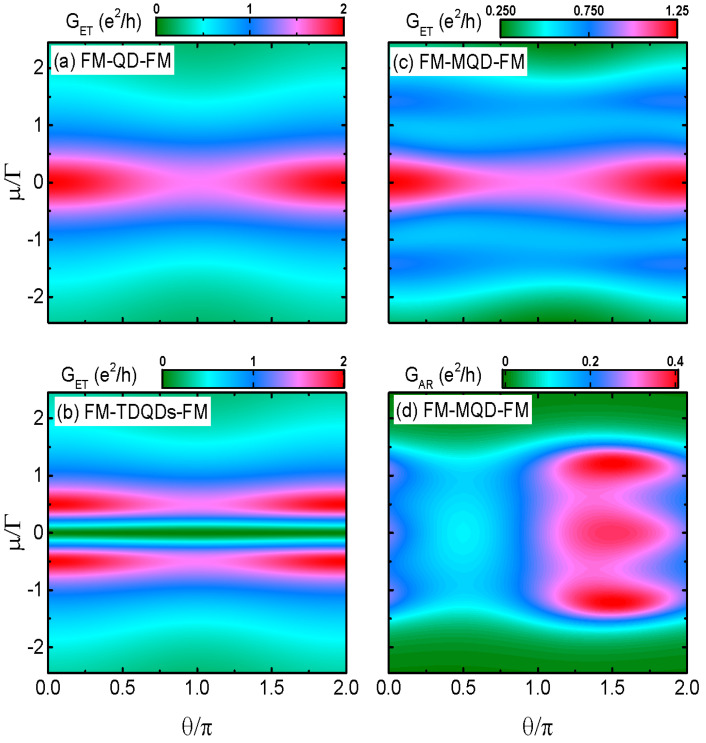
Contour plots of electron tunneling conductance as a function of μ and θ for the cases of FM-SQD-FM in (**a**) and FM-TDQDs-FM in (**b**), in which the AR conductance is zero. Panels (**c**,**d**) are contour plots of the electron tunneling and AR conductances as functions of $\varepsilon_{d}$ and θ in the structure of FM-MQD-FM. In the above figure, we set $\lambda_{0}=\Gamma_{0}$, $P=P_{M}=0.5$, and the other parameters are the same as in Figure 2.

We now study in Figure 4 the impacts of angle θ between the magnetizations of the left and right leads on the transport properties. When the QD is decoupled from the Majorana nanowire (configuration of FM-QD-FM), the line shape of $G_{ET}$ varying with respective to the chemical potential μ at θ=0 in Figure 4a is the same as the black solid line in Figure 2a, which is characterized by a single-peak configuration. With increasing θ from 0 (parallel configuration) to π (antiparallel configuration), $G_{ET}$ is monotonously suppressed, which is the so-called spin valve effect [7,8,23,24]. This is induced by the difference between $\Gamma_{L\sigma }$ (ingoing tunneling rate) and $\Gamma_{R\sigma }$ (outgoing tunneling rate). When the magnetizations on the two leads are in parallel configuration (θ=0), $\Gamma_{L\sigma }=\Gamma_{R\sigma }$, and electrons of both of the two spin components will tunnel resonantly from the left lead to the right one through the QD under the condition of $\mu =\varepsilon_{d}$, and the total conductance reaches its quantum value $2\;e^{2}/h$. For the case of noncollinearity angles, θ=π and the two leads are in antiparallel configuration, the ingoing and outgoing tunneling rates of the two spin directions are $\Gamma_{L↑}=\left(1+0.5\right)\Gamma$, $\Gamma_{R↑}=\left(1-0.5\right)\Gamma$, and $\Gamma_{L↓}=\left(1-0.5\right)\Gamma$, $\Gamma_{R↓}=\left(1+0.5\right)\Gamma$. This imbalance between the tunneling rates induces spin accumulation on the QD and suppresses the conductance amplitude. When the angle changes from π to 2π, the imbalance between the ingoing and outgoing tunneling rates of each spin component is reduced and the conductance is enhanced accordingly. Therefore, in this FM-QD-FM, system the conductance obeys the relationship of $G_{ET}\left(\theta \right)=G_{ET}\left(2\pi -\theta \right)$. As for the FM-TDQDs-FM system, the line shape of the conductance $G_{ET}$ in Figure 4b also resembles that in Figure 3. There are two Breit–Wigner resonances in $G_{ET}$, and the dip at μ=0 is suppressed to be zero. Such behavior of $G_{ET}$ remains unchanged regardless of the value of θ, and it also fulfills the the relation of $G_{ET}\left(\theta \right)=G_{ET}\left(2\pi -\theta \right)$, as in the system of FM-QD-FM.

It is shown in Figure 4c that the resonant peak of ET conductance in FM-MQD-FM resembles those in FM-QD-FM and FM-TDQDs-FM, except that the peak’s height becomes $1.25\;e^{2}/h$, as presented in Figure 2. The behaviors of the satellite peaks are also unchanged by the value of θ. As for the AR conductance in Figure 4c, its line shape is quite different from that of $G_{ET}$, and the triple-peak configuration at θ=0 is changed. Firstly, a dip in $G_{ET}\approx 0.1\;e^{2}/h$ emerges around θ=π/2. Secondly, the triple-peak configuration reappears around θ=3π/2. Thirdly, the peak value around θ=3π/2 is enhanced to about $0.4\;e^{2}/h$, other than the value of $0.25\;e^{2}/h$ for θ=0. Moreover, the AR conductance obeys the relationship of $G_{ET}\left(\theta \right)=G_{ET}\left(2\pi +\theta \right)$. As a result of this, the dependence of the total conductance in FM-MQD-FM on the noncollinearity angle θ is totally different from those in both FM-QD-FM and FM-TDQDs-FM, and it can be used for distinguishing the MBSs from the regular fermionic zero mode.

**Figure 5:**
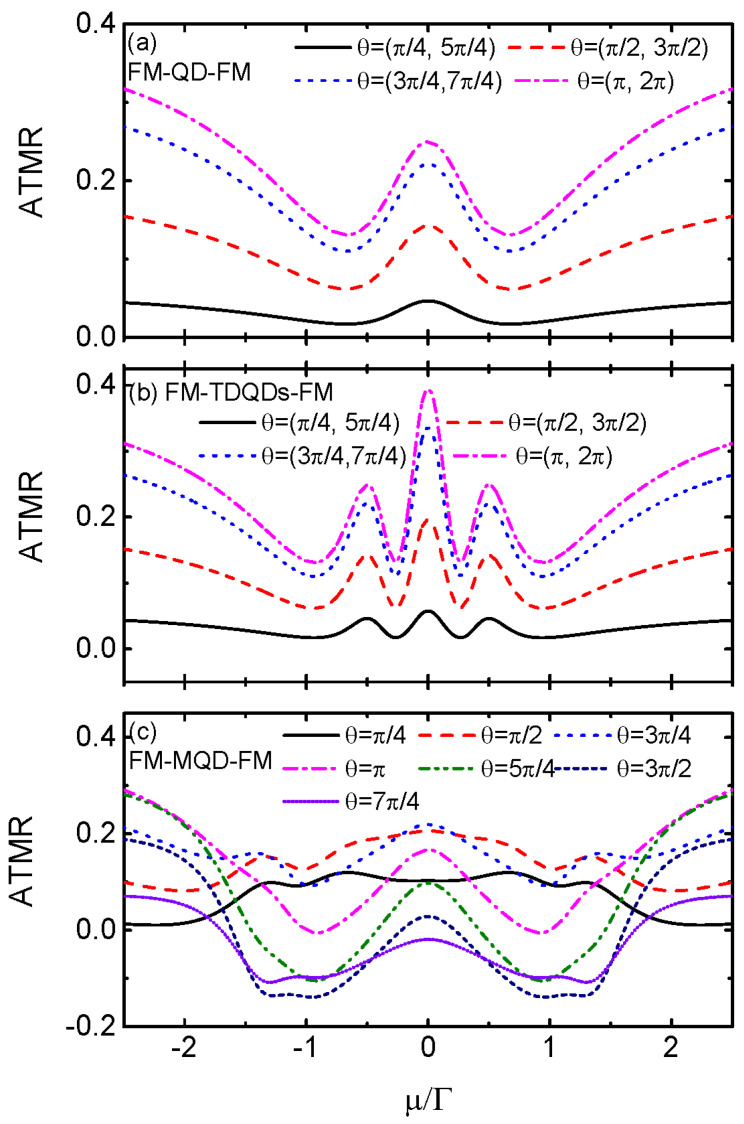
ATMR as a function of the chemical potential μ and different values of θ for the cases of FM-SQD-FM ($\lambda_{0}=0$) in (**a**), FM-TDQDs-FM in (**b**), and FM-MQD-FM in (**c**), respectively. Other parameters are the same as in Figure 4.

Impacts of the MBSs on the transport processes can also be shown via the angle-dependent TMR (ATMR) in addition to the conductances [20,22,23]. Figure 5a presents the ATMR for the case of FM-QD-FM ($\lambda_{0}=0$) varying as a function of chemical potential and different values of the noncollinearity angles. The ATMR is positive regardless of the values of the chemical potential and angle θ, and is characterized by a resonant peak centered at μ=0. It is monotonously enhanced when the angle is changed from 0 to π, and obeys the relationship of ATMR(θ)=ATMR(2π−θ) [20,22,23]. This is because the magnitude of the conductance is monotonously suppressed during this process as compared to the case of θ=0. In the system of FM-TDQDs-FM, the behavior of the ATMR in Figure 5b essentially resembles that of FM-QD-FM, but now the central peak is split into three due to the quantum interference effect. When the QD is coupled to MBS, as shown in Figure 5c, the ATMR depends on the angle θ nonlinearly and can even change its signs. During the chemical regimes of $\mu <|\lambda_{0}|$, the magnitude of the ATMR essentially first increases and then decreases with increasing θ. For θ>π, the ATMR value may change from positive to negative, which serves as a detection mean for the existence of MBSs. Moreover, the relationship between ATMR and the angle is ATMR(θ)=ATMR(2π+θ), which is quite different from that in the cases of FM-QD-FM and FM-TDQDs-FM.

**Figure 6:**
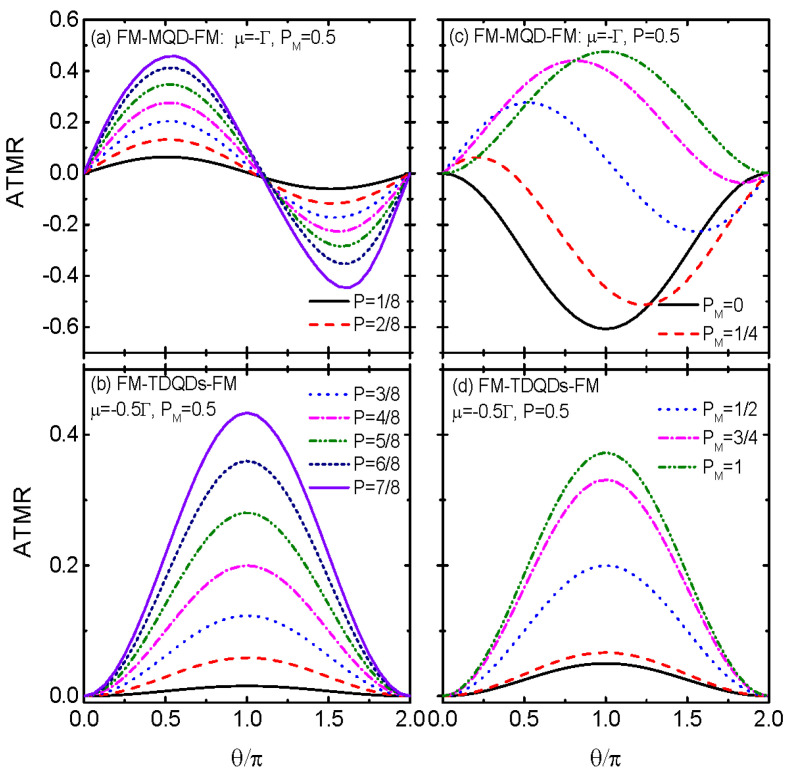
ATMR as a function of the angle θ and different spin-polarization of the leads *P* in (**a**,**b**), and varying $P_{M}$ in (**c**,**d**). Panels (**a**,**c**) show the case of FM-MQD-FM, and (**b**,**d**) are for FM-TDQDs-FM, respectively. Other parameters are the same as in Figure 2 unless listed in the figures.

In Figure 6, we fix $\mu =-\lambda_{0}$, around which obvious negative ATMR emerges, and examine the variation of ATMR with respect to the angle θ. When the QD is coupled to MBS, as shown in Figure 6a, the ATMR oscillates with respective to θ in the form of a sinusoidal function. With increasing spin-polarization *P* of the leads, the magnitude of ATMR is enhanced obviously, with the sinusoidal line shape remaining unchanged. In contrast, the ATMR of FM-TDQDs-FM in Figure 6b shows a broad Breit–Wigner resonance centered at θ=π, and increases monotonously with increasing *P*. The enhancement of the ATMR arises from the increased imbalance of the ingoing and outgoing tunneling rates due to increased spin-polarization of the leads. Figure 6c indicates that the ATMR is negatively characterized by a broad Breit–Wigner resonance at θ=π for $P_{M}=0$ and $\lambda_{↑}=0$, $\lambda_{↓}=\lambda_{0}$, i.e., the MBS couples only to spin-down electrons. With increasing $P_{M}$, the MBS couples to both of the two spin states on the QD, the absolute value of ATMR is suppressed, and the resonance position is changed. Interestingly, the ATMR in the case of $P_{M}=1$ and the MBS coupling only to spin-up state on the QD is totally opposite to that of $P_{M}=0$, i.e., ATMR ${|}_{P_{M}=1}$ = −ATMR ${|}_{P_{M}=0}$. The ATMR for the case of FM-TDQDs-FM in Figure 6d, however, is positive regardless of the value of $P_{M}$ and keeps the single broad peak configuration. Finally, we argue that the present system should be realizable in experiments within current technology, such as by following the general scheme proposed in Ref. [36], in which the common s-wave superconductor may be driven into topological superconductors with MBSs at its ends when it is in contact with noncollinear magnetic textures. Signatures of the MBSs can be demonstrated with the help of scanning tunneling microscopy, which can map the surface topography and probe the local electronic properties of samples with high spectral resolution [39]. Furthermore, functions of the MBSs on the conductance and TMR can be measured via tunneling spectroscopy [39]. In experiments, however, the measured quantity is the differential conductance [39], different to the linear conductance in the present theoretical work. In the linear response regime, i.e., infinitesimal applied bias voltage, they are the same, and the results found here may be experimentally demonstrated. We also emphasize that in the present paper, only the linear conductance and TMR are studied. In the presence of finite bias voltage and the system being driven into nonequilibrium states, the spin-dependent current and the corresponding TMR may even be interesting and are worth being studied in the future.

## 4. Summary

In summary, we studied electronic transport through a QD sandwiched between two ferromagnetic leads with noncollinear magnetizations. We find that when the QD is additionally side-coupled to a superconductor nanowire hosting MBSs at its ends, the linear conductance and ATMR are significantly changed. When the MBS couples simultaneously to both of the two spin states on the QD, the spin-up and spin-down resonant conductance peak value is $0.75\;e^{2}/h$, which is different to the $0.5\;e^{2}/h$ previously found when the MBS is coupled to only one spin state.If the QD is coupled to regular fermonic zero mode and the Fermi level is aligned to the dot level, however, the conductance is suppressed to be zero. We find that the conductance and ATMR in the presence of dot–MBS coupling depends on the noncollinearity angle in quite a different way to the case of the QD interacting with regular fermonic zero mode. It is also found that both the magnitude and sign of the ATMR can be adjusted by the dot–MBS coupling strength and its spin-polarization. Such a sign change of the ATMR can hardly emerge in the system of the QD coupled to regular fermonic zero mode, where the ATMR remains positive and is characterized by a broad Breit–Wigner peak. It should be noted that possible functions of intradot Coulomb interaction and MBS–MBS hybridization on the conductance and TMR are neglected in the present paper, and should be considered in the future since they induce interesting transport processes and change the above two physical quantities.

## Data Availability

All data included in this study are available upon request by contact with the corresponding author.
